# A Novel High-Speed Split-Gate Trench Carrier-Stored Trench-Gate Bipolar Transistor with Enhanced Short-Circuit Roughness

**DOI:** 10.3390/mi15060680

**Published:** 2024-05-22

**Authors:** Zhehong Qian, Wenrong Cui, Tianyang Feng, Hang Xu, Yafen Yang, Qingqing Sun, David Wei Zhang

**Affiliations:** 1 School of Microelectronics, Fudan University, Shanghai 200433, China; 2 Jiashan Fudan Institute, Jiaxing 314100, China

**Keywords:** CSTBT, split-gate technology, high speed, short-circuit roughness

## Abstract

A novel high-speed and process-compatible carrier-stored trench-gate bipolar transistor (CSTBT) combined with split-gate technology is proposed in this paper. The device features a split polysilicon electrode in the trench, where the left portion is equipotential with the cathode. This design mitigates the impact of the anode on the trench gate, resulting in a reduction in the gate-collector capacitance (C_GC_) to improve the dynamic characteristics. On the left side of the device cell, the P-layer, the carrier-stored (CS) layer and the P-body are formed from the bottom up by ion implantation and annealing. The P-layer beneath the trench bottom can decrease the electric field at the bottom of the trench, thereby improving breakdown voltage (BV) performance. Simultaneously, the highly doped CS layer strengthens the hole-accumulation effect at the cathode. Moreover, the PNP doping layers on the left form a self-biased pMOS. In a short-circuit state, the self-biased pMOS turns on at a certain collector voltage, causing the potential of the CS-layer to be clamped by the hole channel. Consequently, the short-circuit current no longer increases with the collector voltage. The simulation results reveal significant improvements in comparison with the conventional CSTBT under the same on-state voltage (1.48 V for 100 A/cm^2^). Specifically, the turn-off time (t_off_) and turn-off loss (E_off_) are reduced by 38.4% and 41.8%, respectively. The short-circuit current is decreased by 50%, while the short-circuit time of the device is increased by 2.46 times.

## 1. Introduction

Currently, there is an urgent need for higher-speed, lower-cost, and higher-efficiency applications. The insulated-gate bipolar transistor (IGBT) stands out as a globally representative product of the third technological revolution in power electronics. As a composite full-controlled voltage-driven power semiconductor device, the IGBT exhibits excellent performance, with advantages such as a low cost, relatively large current, and easy switching, meaning that it forms the core of power converters [1,2,3,4,5].

Existing IGBTs can be roughly divided into planar-gate IGBTs and trench IGBTs, according to the characteristics of the gate electrode. Trench IGBTs have been shown to be competitive candidates for IGBT power applications, due to their superiority in terms of manufacturing, reliability, current handle capability, and cell density [6,7,8,9,10,11]. In planar-gate IGBTs, in the forward conduction state, the PN junction formed by the P-well and the N-drift region is in a slightly reverse-biased state. Consequently, a space-charge region with a certain width is formed, which occupies a certain space and increases the impedance in the current path [12,13,14,15]. Compared with the planar-gate IGBT, the trench IGBT can greatly reduce the turn-on voltage drop without increasing the turn-off loss (E_off_). Trench IGBTs also eliminate the JFET effect, increase the channel density, and enhance the carrier concentration near the surface to improve the turn-on voltage (V_on_) [16,17,18,19,20]. And an important development direction represented by CSTBT is IGBT surface structure design technology. Through the special surface structure design, the breaking voltage and anti-interference ability of the device can be improved, and the leakage current of the device can be reduced, so as to reduce the positive conduction voltage drop and reduce the loss during turn-off.

However, conventional shield-gate trench IGBTs (including CSTBTs) face two crucial challenges. Firstly, the electric field at the bottom of the trench is too concentrated, resulting in early breakdown at the bottom, and the breakdown voltage of the device is weakened. At the same time, the breakdown characteristics of the device are very sensitive to the trench depth and doping concentration near the bottom. Secondly, due to the existence of the Miller effect, the discharge of the trench capacitor is usually referred to as the part with the largest energy consumption in the entire discharge process. A tradeoff relationship exists between V_on_ and E_off_, relying on trench size. Generally, a deeper trench can increase the channel density in the on-state of the device, achieving a smaller V_on_. However, the gate-collector capacitance (C_GC_) increases significantly as the trench depth increases. As a result, the dynamic characteristics of the device deteriorate, including the turn-off time (t_off_) and E_off_. Some approaches have been proposed to address C_GC_ reduction for trench IGBTs, primarily focusing on the lower portion of the trench. These methods include implementing thick oxide to the trench bottom or, optionally, to the sidewalls [2,3,4,5]. Nonetheless, these methods suffer from poor process compatibility and the great impact of process variation on the device performance. At the same time, with the increase in power density in the IGBT, short-circuit characteristics are becoming more and more important and many new structures have been proposed [18,19]. If the short-circuit current density of the IGBT is too high, then the safe operating area (SOA) of the device becomes narrow in high-voltage and high-current applications. At present, the main solutions include reducing the effective channel density, which leads to an increase in conduction loss. The self-biased split-gate pMOS is thought to be an effective solution to this problem; however, currently similar structures suffer from poor process compatibility and the great impact of process variation on device performance.

To solve the problems of excessive turn-off loss and poor short-circuit tolerance in IGBT devices, this study introduces the design of a novel split-gate trench CSTBT, demonstrated by numerical simulations. This design features low V_on_, low E_off_, a low saturation current, and enhanced short-circuit capability. Notably, the device performance is unaffected by the deviation in the split-gate process.

## 2. Structure and Process Flow

**Figure 1:**
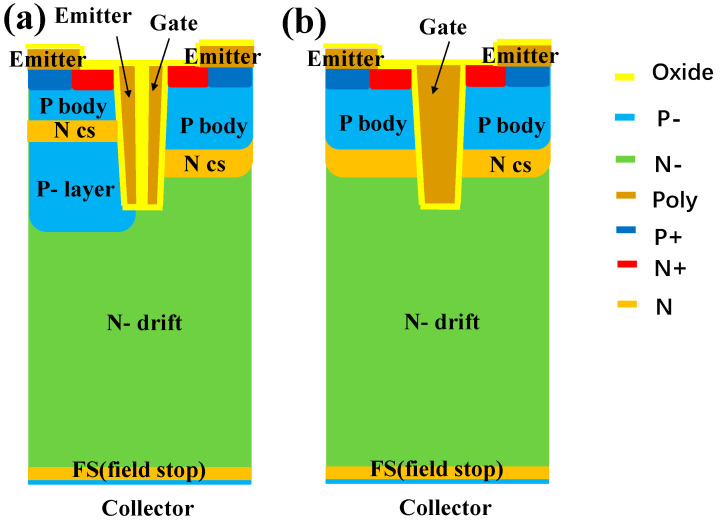
(**a**) The proposed SGT-CSTBT and (**b**) conventional CSTBT.

The schematic diagram of the proposed split-gate CSTBT is illustrated in Figure 1a, where the polysilicon electrode within the trench is divided into two segments: the split electrode on the left side is linked to the emitter cathode, while the split electrode on the right side functions as the conventional gate electrode. On the left side of the device cell, the P- layer, the carrier-stored (CS) layer, and the P-body are formed from the bottom upwards by ion implantation and annealing processes.

The proposed SGT-CSTBT has the same doping profile as, and a similar structure to, the traditional structure, except for the split-gate structure. The right part includes the P-body and the identical doped CS layer. The drift zone thickness, doping concentration, and collector region doping remain identical, to ensure control variable. Major structural parameters are shown in Table 1.

**Figure 2:**
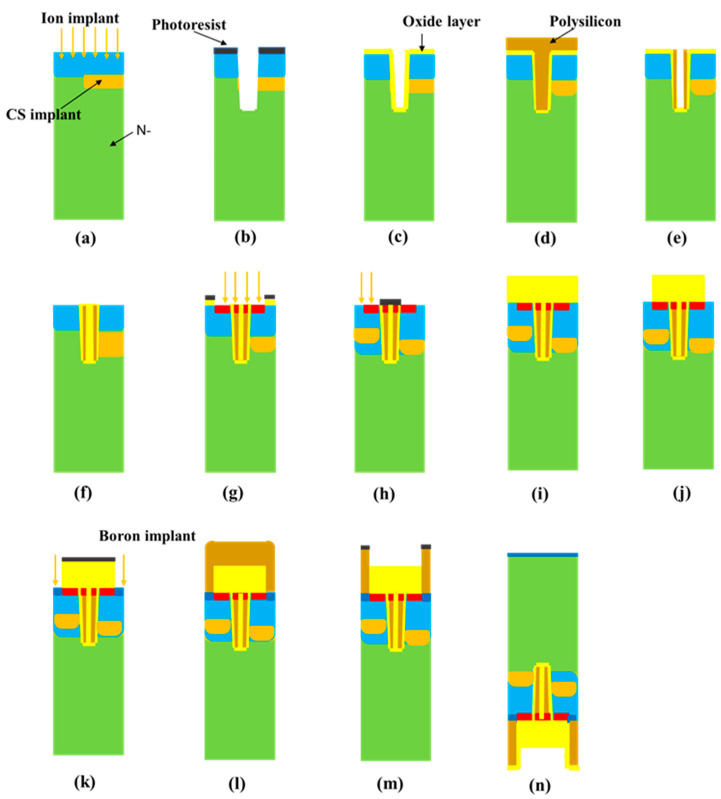
Main process in the proposed split-gate device.

The general process flow of the device is shown in Figure 2 and mainly includes the following: (a) CS-layer ion implantation; (b) deep trench etching, (c) dielectric oxide layer growth; (d) polysilicon deposition; (e) polysilicon etching; (f) oxide layer etching; (g) N+ source area implantation and surface passivation layer deposition; (h) left higher doping CS-layer injection; (i) surface passivation; (j) surface oxide etching; (k) source area P+ implantation; (l) surface passivation; (m) source metallization; (n) back-collector ion implantation and metallization.

Taking into account the variation in the actual process, the deposition and etching rates of polysilicon is unstable, and the thickness of the polysilicon with the two separate electrodes on each side may be not a fixed value. The separation gate electrode devices with polysilicon thickness of 200 nm, 300 nm, 400 nm, 500 nm, and 600 nm are simulated, and device performances are compared.

**Figure 3:**
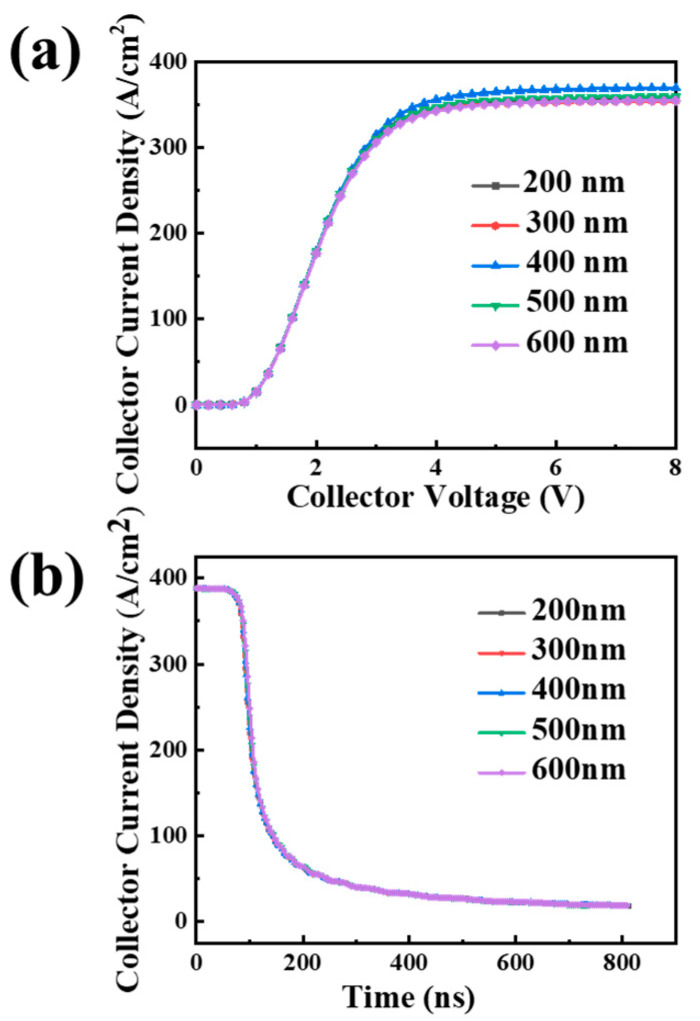
(**a**) Output characteristics of SGT-CSTBT with different separation polysilicon gate electrodes and (**b**) turn-off waves with different separation polysilicon gate electrodes.

The output characteristic curve and the turn-off characteristic curve are depicted in Figure 3. The output characteristic curves of the devices with different separation electrode thicknesses almost overlap, as do the turn-off characteristic curves, which means that the variation in the polysilicon thickness hardly affects the output characteristics (V_on_ and Saturation current) and turn-off characteristics (E_off_) of the device.

These results show that the device has a high degree of insensitivity to the variation in the split-gate trench formation process.

## 3. Simulation Results and Discussion

The device model was established by the Sentaurus Technology Computer-Aided Design (TCAD) simulation, and the process conditions are based on the SMIC 0.18 µm process node.

**Figure 4:**
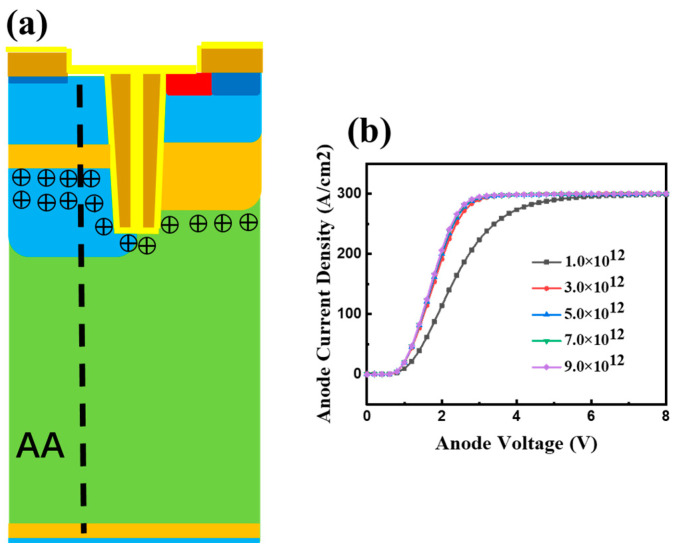
(**a**) Working mechanism of the device and (**b**) output characteristics of the device with different left-CS-layer doping.

As shown in Figure 4a, the existence of the highly doped CS layer on the left increases the resistance of the holes traveling from the N-drift region to the emitter, simultaneously elevating the carrier concentration near the emitter side. This CS layer ensures that the device maintains a low V_on_ drop and substantially reduces the t_off_.

**Figure 5:**
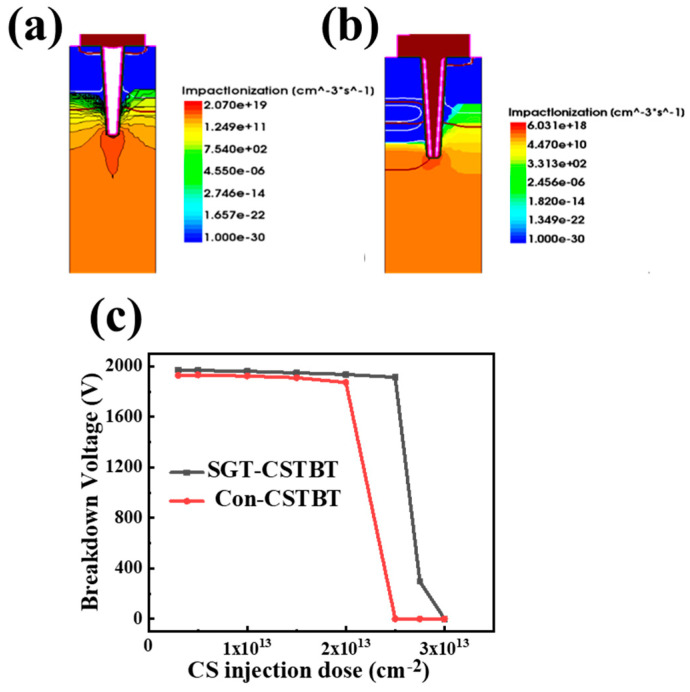
(**a**) Impact ionization rate of conventional CSTBT with 1900 V reverse bias. (**b**) Impact ionization rate of SGT-CSTBT with 1900 V reverse bias. (**c**) Relationship of the BV and V_on_ with CS injection dose.

Simulation results show that an increase in the CS doping concentration on the left leads to a reduction in V_on_. As shown in Figure 4b, the V_on_ of the proposed device decreases with the increase in CS-layer injection dose on the left. This is attributed to the fact that a higher doping concentration of the CS layer on the left can enhance the hole-carrier storage effect. When the injection dose of the left CS layer reaches 9.0 × 10^12^/cm^2^, further increasing the injection dose has less effect on reducing V_on_ in Figure 4b. The influence of the left CS layer on the increase in the hole accumulation effect reaches saturation. At the same time, the results show that, within the injection dose range from 3.0 × 10^12^/cm^2^ to 9.0 × 10^12^/cm^2^ for the left CS layer, the breakdown characteristics of the device remain constant. The V_on_ of the proposed SGT-CSTBT and conventional CSTBT is compared in Figure 5c. It is clear the proposed SGT-CSTBT achieves a lower V_on_ for a current density of 100 A/cm^2^, with both devices have the same doping profile on the right, and the left CS-layer injection dose is 3.0 × 10^12^/cm^2^.

Compared to conventional CSTBT, a P-type buffer layer is introduced at the bottom of the trench to reduce the electric field, which helps to lower the impact ionization rate at the bottom of the trench by about 70%. This reduction in impact ionization allows for a more heavily doped CS layer, enabling a decrease in the on-state voltage without significantly affecting the breakdown capability.

By comparing the breakdown characteristics of conventional CSTBT and SGT-CSTBT, it can be seen that in Figure 5, with the same doping dose, the proposed SGT-CSTBT consistently demonstrates a higher breakdown voltage (BV). Specifically, as the doping dose increases, the BV of the SGT-CSTBT remains near 2000 V, while the BV of the conventional CSTBT drastically drops to 0 V when the injection dose exceeds 2.0 × 10^13^ cm^−2^. This highlights the improved breakdown performance of the SGT-CSTBT design compared to the conventional CSTBT in terms of maintaining a higher BV, even at higher injection doses.

**Figure 6:**
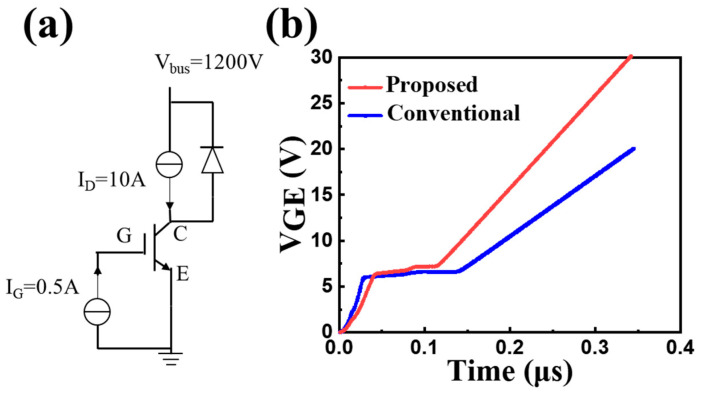
(**a**) Gate-charging test circuit and (**b**) gate-charging curve.

The Miller effect greatly increases the switching time and switching loss of the device. The value of C_GC_ is usually used as the key basis for evaluating the E_off_ of the device, and the size of C_GC_ can be inferred by the length of the plateau period of V_GE_ in the stage. Figure 6. shows a gate charge test circuit and gate charge curve for both CSTBTs. First, the gate voltage increases linearly, which charges up the capacitance C_GE_. Then, the gate voltage remains almost constant within a period of time, charging up the Miller capacitance.

The charging time of the proposed SGT-CSTBT for the Miller capacitance decreases by 40.8%, compared with the conventional CSTBT. The Miller capacitance of the proposed SGT-CSTBT, equivalently, decreases from 8.523 to 4.81 nF/cm^2^. The cathode-connected gate in the trench can eliminate the impact of the anode on the trench gate to some extent. And the gate-collector capacitance (C_GC_) of the proposed device decreases as the coupling area between the trench and anode is greatly reduced. Therefore, a better dynamic performance is achieved.

**Figure 7:**
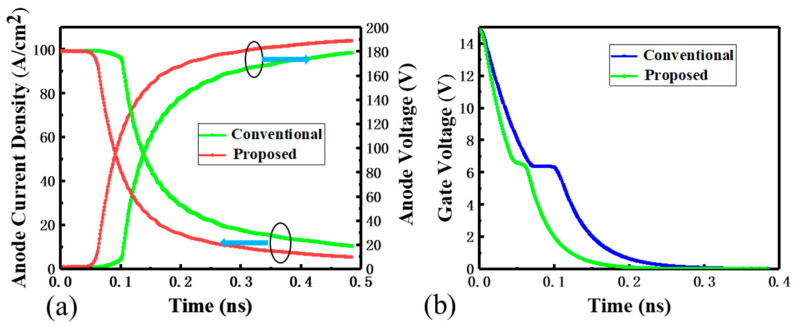
(**a**). Turn-off waves under inductive load in the conventional CSTBT and SGT-CSTBT with the same V_on_ (Vcc = 1200 V Lc = 1.5 mH Rg = 10 ῼ Ci = 50 pF). (**b**) Gate voltage waves during turn-off.

Figure 7a depicts the turn-off waves under the inductive load for both devices with the same V_on_. The turn-off time is defined as the time from the t_1_ (V_G_ = 13.5 V) to t_2_ (J_anode_ = 10 A/cm^2^). Obviously, the turn-off time of the SGT-CSTBT is shorter than that of the conventional CSTBT. The turn-off time of the SGT-CSTBT achieves a 38.4% reduction, which indicates an excellent E_off_ performance in the SGT-CSTBT. Additionally, the proposed SGT-CSTBT has a smaller tail current at the current falling stage of the turning-off. This reduction is attributed to the lighter-doped P-collector (by adjusting the doping of the P-collector to make the V_on_ consistent). As mentioned earlier, in the turn-off state, the Miller effect is effectively suppressed, as depicted in Figure 7b. And the turn-on loss and turn-on time are also optimized, mainly due to the reduction in Miller capacitance.

**Figure 8:**
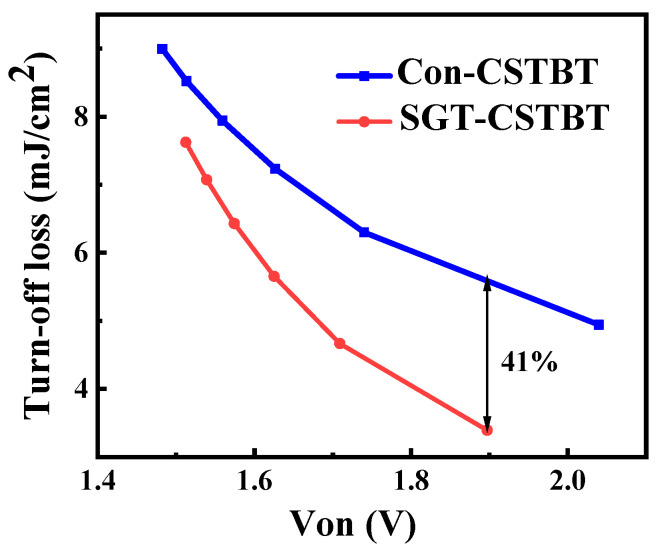
Trade-off relationships between V_on_ and E_off_ (obtained by varying the doping concentration of the P emitter at the anode side).

Figure 8 illustrates the relationship between the E_off_ and V_on_ of the two different structures after changing the peak P-collector doping concentration. Under E_off_ = 5 mJ/cm^2^, the V_on_ of the SGT-CSTBT is 1.68 V, which is 17.5% lower than the 2.04 V of the Con-CSTBT. As V_on_ changes from 1.5 V to 2.0 V, the proposed device can effectively reduce the E_off_ as expected. Under V_on_ = 1.9 V, the E_off_ of the SGT-CSTBT is 3.3 mJ/cm^2^, which is nearly 41.8% lower than the 5.67 mJ/cm^2^ of the Con-CSTBT. It is clear the proposed SGT-CSTBT can significantly improve the trade-off relationship, as expected.

**Figure 9:**
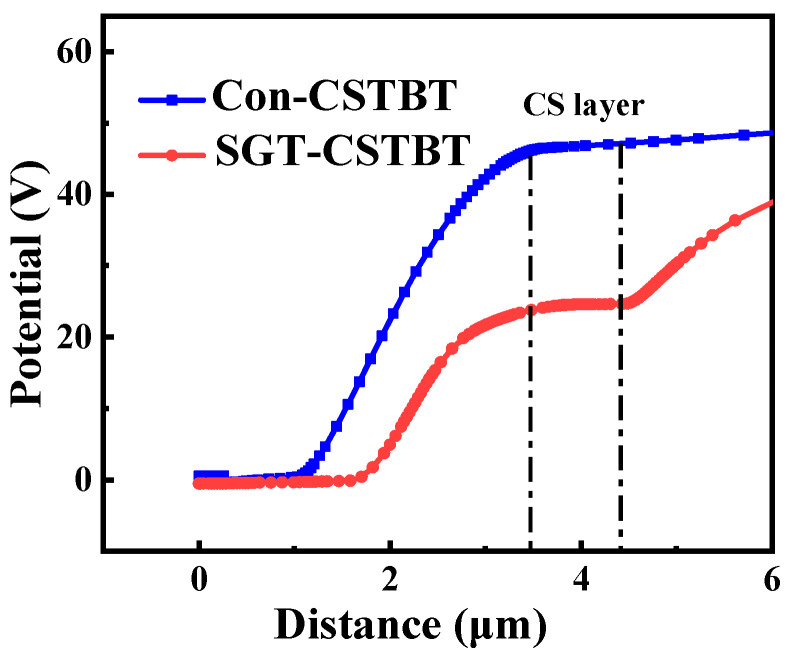
The vertical potential distribution (V_G_ = 15 V, V_C_ = 100 V).

**Figure 10:**
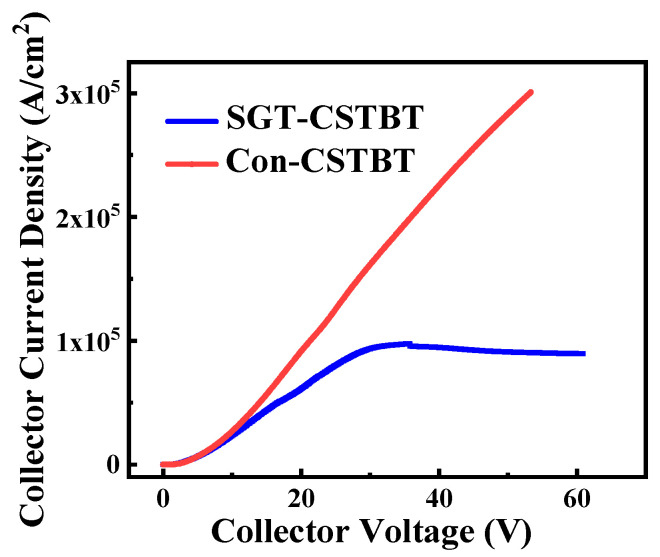
Short-circuit current at different collector voltages.

The potential distribution of the proposed SGT-CSTBT and conventional CSTBT with 100 V anode voltage (V_G_ = 15 V) are compared in Figure 9. It is clear that V_CS_ (the potential of the N-layer) of the proposed SGT-CSTBT is much lower than that of the conventional device. And when the voltage of the anode exceeds 35 V, the short-circuit current of the proposed device no longer increases and even appears to decrease, as shown in Figure 10. The reason for the enhanced short-circuit roughness of the device is that, with the increase in collector voltage, the potential of the left CS layer increases. When the potential of the second P-type base reaches the threshold voltage of the PMOS, the PMOS is turned on, and the excess holes are discharged from the PMOS channel, so the potential of the second P-type base no longer increases.

The short-circuit current simulation results show that the proposed SGT-CSTBT structure is obviously optimized under different short-circuit voltages, as shown in Figure 10. The short-circuit current is reduced by 50% at a 40 V short-circuit voltage.

**Figure 11:**
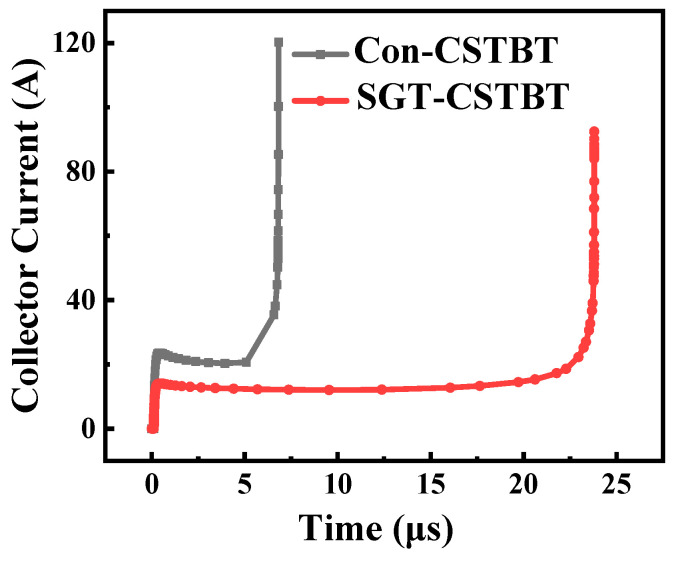
Simulated short-circuit failures in the conventional CSTBT and SGT-CSTBT.

**Figure 12:**
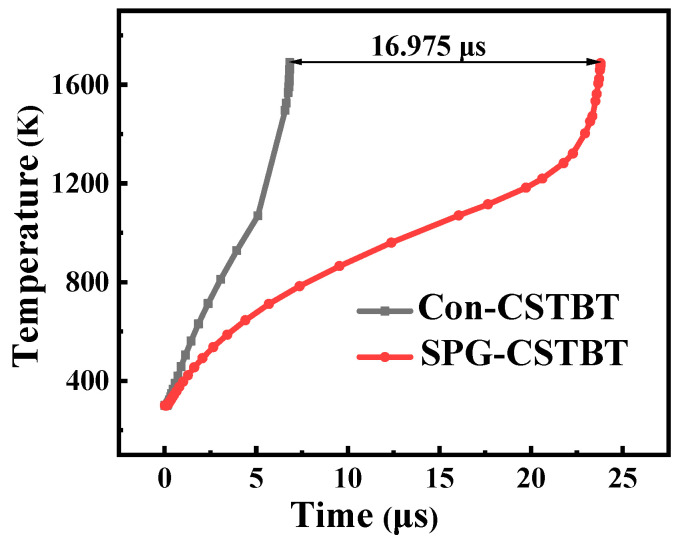
Maximum device temperature curves in the short-circuit situation of CSTBT and SGT-CSTBT. The self-heating effect is considered by the LAT.TEMP model, with the initial device temperature of 300 K.

Figure 11 and Figure 12 illustrate short-circuit waves. They indicate that the SGT-CSTBT withstands a significantly longer short-circuit duration time (the device failure time is defined when the lattice temperature reaches 1687 K) than that of the conventional CSTBT before failure. Owing to the smaller saturation current density, the short-circuit safe operating area (SCSOA) of the proposed SGT-CSTBT structure is about 2.46 times larger than that of the conventional CSTBT structure.

## 4. Conclusions

A novel split-gate CSTBT is proposed and investigated via numerical simulations. Using a combination of the hole-channel and carrier-stored technologies, the SGT-CSTBT presented has excellent performance. t_off_ is reduced without sacrificing the conduction loss and V_on_. The P channel is closed in on state, servicing as a hole-stop layer to enhance the accumulation of holes near the cathode. During the turn-off process, this P channel provides an additional low resistance path for the hole current and reduces the t_off_. In the blocking state, the P layer beneath the trench can significantly alleviate the trade-off of the BV- and CS-layer concentrations. And the short-circuit duration time increases by 246%.

## Figures and Tables

**Table 1:** Major structural parameters.

Parameter	Value
Cell pitch	4 μm
Gate oxide thickness	120 nm
N-drift doping	5.9 × 10^13^ cm^−3^
CS left doping	1.0 × 10^18^ cm^−3^
CS right doping	2.0 × 10^17^ cm^−3^
P-well doping	3.0 × 10^16^ cm^−3^
Trench depth	5 μm

## Data Availability

Data are included within the article.
